# Prevalence of tobacco use and associated risk factors among pregnant women in Maracha District, Uganda

**DOI:** 10.5897/jphe2020.1276

**Published:** 2021-04-30

**Authors:** John Bosco Alege, Russall Okudra Jurua, Judith Drazidio

**Affiliations:** 1 Department of Environmental Health, Maracha District Local Government, Uganda; 2 Institute of Public Health and Management, Clarke International University, Kampala Uganda; 3 Schools of Public Health and Applied Human Sciences, Kenyatta University, Nairobi, Kenya

**Keywords:** Prevalence, tobacco use, risk factors, pregnant women

## Abstract

Globally, tobacco use has become the largest public health threat that kills around 7 million people annually, of which about 6 million deaths are due to direct tobacco use, and 890,000 are attributed to passive smoking. This study assessed prevalence and associated risk factors of tobacco use among pregnant women, 15 to 49 years. Health facility-based analytical cross-sectional study was conducted among 199 pregnant women using purposive sampling technique and convenient sampling technique for the respondents. Chi-square test and binary logistic regression were used to compare quantitative data at a 95% CI. Prevalence of tobacco use among respondents was 39.2%. The results gives those who starting to smoke at more than 30 years (p≤0.001), agreeing that smoking makes pregnant women feel they have total control over their health and life (p≤0.008); the likelihood of tobacco use reduced among pregnant women aged 20-29 years (p≤0.032), those disagreeing that tobacco use as a sign of maturity (p≤0.003) and disagreeing that smoking can help calm nerves, control moods, and alleviates stress (p≤0.002). However, cultural factors that reduced the chances of smoking in pregnancy include smoking more than five times a day (p≤0.01) and smoking cigarettes (p≤0.017), were statistically associated with smoking. High prevalence of tobacco use among pregnant women in Kijomoro and Eliofe health center III was recorded. Thus, there is need to sensitize pregnant women about tobacco-related health problems on them and their unborn children.

## INTRODUCTION

Maternal smoking, especially during pregnancy, has adverse consequences for both mother and child as it is the strongest predictor of fetal outcomes as well as ectopic pregnancy and orofacial clefts (Murthy and Mishra, 2017) Besides, tobacco use among pregnant women increases risks of infertility, increased risks of spontaneous abortions, premature delivery, stillbirths, and having children with low birth weight. Other complications include long term effects on surviving, children such as long term disabilities like cerebral palsy (MoH, 2013). Other complications like placenta Previa placental abruption, and pre-eclampsia, and poor fetal outcomes varying from low birth weight, overall perinatal mortality, stillbirth, and preterm birth as well as miscarriages (Murthy and Mishra, 2017). There has been a global effort to prevent tobacco use like tobacco taxes, tobacco advertising bans, warning labels, and some free control. Despite these, the tobacco epidemic is accountable for about 5.4 million global deaths annually (WHO, 2013). Moreso, tobacco-related deaths among women aged 20 years and above are anticipated to rise from 1.5 million in 2004 to 2.5 million by 2030 and 75% deaths were expected to occur in low and middle-income countries (WHO, 2009). Meanwhile, Sub-Saharan Africa has the lowest rate of cigarette consumption due to low purchasing power and cigarette use (Achia TNO, 2015). However, many African countries lack reliable data on tobacco use by women especially pregnant women, which are needed to inform tobacco control and prevention efforts (Rani, 2003).

In Uganda, the global Tobacco Adult survey indicates that 7.9% of them aged 15 to 49 years had used tobacco products, of which 11.6% were men and 4.6% women (MoH, 2013). Furthermore, Uganda Tobacco Control Act (UTCA) (2015) Part III provides the right of every person to a tobacco-free environment. The same act stipulates that a person consuming a tobacco product should ensure that he or she does not expose another person to tobacco smoke. Additionally, Article 39 of the 1995 Constitution of the Republic of Uganda guarantees “citizens’ right to a clean and healthy environment. All these imply that tobacco use by pregnant women is prohibited either by direct or passive smoking (UTCA, 2015). Despite this fact, Uganda demographic and health survey report (2016), indicates that the prevalence of tobacco use among pregnant women in the West Nile region was 24% compared to 3% in Bukedi and 4% in the Bugishu region eastern Uganda. However, the associated risk factors for tobacco use among pregnant women in Maracha County remain unclear. This study assessed the prevalence and associated risk factors of tobacco use among pregnant women in Maracha district.

## METHODOLOGY

### Study area

This study was conducted at Kijomoro and Eliofe health center III Maracha district. Maracha district is found in the northwestern region that lies 560km north of Kampala on the Arua-Koboko highway and shares borders with Yumbe in the northeast, Koboko to the north, the democratic republic of Congo to the west, and Arua to the south and east. The district has a total area of 441 square km and is home to 186176 (2014 census) people who reside within the county.

### Study design, study population and sample size determination

The study used a health facility-based analytical cross-sectional study design to describe and analyze the prevalence and associated risk factors to Tobacco use among pregnant women attending health care services in Kijomoro and Eliofe health center III Maracha district. The study population comprised of pregnant mothers aged 15 to 49 years attending antenatal care services at Kijomoro and Eliofe health center III. The sample size was determined using Krejcie and Morgan table (2006) at a 95% confidence interval with a 10% non-response rate. According to health facility record 2018, about 400 women attend antenatal care services in the past months and the sample corresponding to this population is 196. Considering a 10% non-response rate, 216 respondents were expected to participate in this study.

### Sampling and data collection technique and data collection instrument

Purposive non-probability sampling technique was used to select Kijomoro and Eliofe health center III. These facilities were selected because they offer antenatal care services to pregnant women and are based on the availability of ANC services (Maternity Unit) for pregnant mothers aged 15-49 years. Thereafter, a convenient sampling procedure was used to select study participants, which means that all pregnant women in the required age category at present at the health facility during the time of the survey, without any other health condition were enrolled in the study. Before the commencement of the actual data collection process, research assistants were trained to ensure that they understand the purpose of the study as well as keep respondent’s information used for the purpose of the study confidential. However, data collection tools were pretested in Oleba health center III in Maracha County to ensure questions were clear and allowed the inclusive gathering of the required data regarding prevalence and associated risk factors with Tobacco use among pregnant women. The questions which had ambiguity during pre-test were revisited and modified as required; hence questions were added to cater to the information needed for the study. Finally, the questionnaire was translated into the local language (Lugbara) before the actual data collection process.

The data was collected using the researcher administered technique through face-to-face interaction with each respondent. A structured questionnaire comprising of: socio-demographic characteristics maternal age, ethnic group, place of residence, religion, marital status, level of education, parity, family size, occupation, income levels, self-esteem, and self-image. Also, cultural factors of tobacco use such as social acceptance, social cohesion, social norms, peer influence, parental influence, and partner influence were used to collect the required data regarding the prevalence of tobacco use and associated risk factors among pregnant women.

### Data management and analysis procedure

The quantitative data was entered in EPIdata version 3.1 and then records were exported to statistical package for social sciences version 23.0 for analysis. The dependent variable was assessed whether a respondent has smoked during the current pregnancy and was categorized as 1-yes and 2-no. The independent variables were demographic characteristics of the respondents and socio-cultural factors associated with tobacco use among pregnant mothers. Frequencies and percentages were used to describe the characteristics of the pregnant women at Univariate analysis. A Chi-square test was performed to establish the level of association between tobacco use and independent variables at a 95% confidence level, while fisher exact test was used to determine the level of association for cell counts less than five observations. However, to determine the existence and strength of association between dependent and independent variables, the binary logistic regression model was fitted at 95% and results were presented using both crude and adjusted odds ratios. Variables whose odds ratio were greater than one (aOR>1) were considered statistically significant to tobacco use among pregnant women while those whose odds ratio was less than one reduced the chance of tobacco use during pregnancy.

### Ethical considerations

Ethical approval was obtained from the Research and Ethics Committee of Clarke International University (UG-REC). Upon ethical approval, administrative permission was obtained from the Maracha district health department and in-charge of the Kijomoro and Eliofe health facility. Throughout the study period, all the respondents voluntarily consented either verbally or written informed consent to be enrolled as study participants. Also, the confidentiality of respondent information was ensured throughout the study, as the respondent’s particulars were not captured.

## RESULTS

### Prevalence of tobacco use among respondents

A total of 199 pregnant women were enrolled in this study, of these 39.2% (N=) had used tobacco in their current pregnancy. Despite the current smoking during pregnancy, 19.1% of the pregnant women had ever smoked in their life, 14.6% had smoked for 5-15 years, 7% 16-25 years, 9.5% 26-35 years, 3.5% 36-45 years while 4.5% had smoked for 46 years and above. However, the pregnant women used to smoke tobacco (19.6%). Among the respondents, 20.1% smoked more than five times a day as compared to 19.1% who smoked less than five times a day (Table 1).

### Demographic characteristics of the respondents

The study finding revealed that almost half 99 (49.7%) of the respondents were aged 20-29 years, while less than a quarter 36 (18.1%) were aged 15-19 years. However, age had a significant association with tobacco use (p≤0.021). Besides, slightly more than half 138 (69.3%) of the respondents married at age 15-19 years, while only 5 (2.5%) married at more than 30 years and this showed statistical significance with tobacco use (p≤0.001). Furthermore, more than half 126 (63.3%) of the respondents were Catholics, while the least 7 (3.5%) belonged to other religious denominations (p≤0.004). Meanwhile, the study finding indicated that the majority of 179(89.9%) of the respondent were married while 4(2%) were cohabiting, 6(3%) single and had divorced (p≤0.001). Similarly, 150(75.4%) of the respondents had attained primary education, while 2(1%) had a university education, 10(5%) tertiary education, and no formal education (p≤0.007).

The study showed that current pregnancy order had a significant association with tobacco use in pregnancy (p≤0.024), while the proportion indicates that 39(19.6%) of the respondents had more than five pregnancies unlike 23(11.6%) that had fifth pregnancy. Besides, the number of children ever born to a woman significantly influenced tobacco use in pregnancy (p≤0.004). However, 32(16.1%) of the respondents never had children, while 48(24.1%) had one child, 29(14.6%) five children and above, and 22(11.1%) four children. Also, number of children living per woman had a significant association with tobacco use during pregnancy (p≤0.024). Meanwhile, 47(23.6%) of the respondents agreed that they had one child living, while 19(9.5%) had four children.

When respondents were asked about their opinion towards tobacco use in pregnancy, a quarter of 50(25.1%) of them agreed that tobacco use is a sign of attaining maturity, while 97(48.1%) disagreed and 52(26.1%) were not sure. Despite this fact, the study established a significant association with using tobacco as a sign of maturity in pregnancy (p≤0.009). Moreso, the study found that only 18(9%) of the respondents agreed that tobacco use in pregnancy is a sign of wealth, more than half, 137(68.8%), disagreed and 44(22.1%) were not sure (p≤0.034). However, 28(14.1%) of the respondents agreed that they use tobacco to have control over their healthy life, 130(65.3%) of them disagreed; while 41(20.6%) were not sure (p≤0.02). Finally, 43(21.6%) of the respondents agreed that tobacco use help to calm nerves, more than half 119(59.8%) disagreed, and 37(18.6%) were not sure (p≤0.001) (Table 2).

### Factors associated with tobacco use among pregnant women

In the unadjusted logistic regression model, tobacco use during pregnancy increased with age at the start of smoking, especially starting at 20 to 29 years (cOR=21.9; 95%CI: 4.688-103.235,p≤0.001). Starting to use tobacco more than 30 years and above increased chances of smoking during pregnancy by 38 times as compared to their counterparts (cOR=38; 95%CI: 12.172-119.227, p≤0.001). Besides, having agreed that tobacco use in pregnancy is a sign of attaining maturity increased chances of its use by 1.2 times likely (cOR=1.216; 95%CI: 0.522-2.837, p≤0.65), while having disagreed reduced chances of tobacco use 3.8 times likely (cOR=3.841;95%CI:1.4-10.537,p≤0.009). The chances of tobacco use among pregnant women were found higher among respondents who smoke to have control over their health (cOR=6.24;95CI:2.12-18.368,p≤0.001). Meanwhile, respondents who agreed that tobacco use help to calm nerves were three timely to use tobacco during pregnancy (cOR=3.838; 95%CI:1.615-9.12, p≤0.002). While, respondents who disagreed that tobacco use help to calm nerves had eight times reduced chances of using tobacco during pregnancy (cOR=8.908;95%CI:2.43-32.663,p≤0.001). However, among the cultural factors, using tobacco products accepted by cultures such as cigarettes and snuff had increased chances of tobacco use (cOR=20.25;95CI:1.878-218.39,p≤0.013) and (cOR=23.707; 95%CI: 2.912-193.025, p≤0.003) respectively.

The study revealed that using both smoking and smokeless form of tobacco had reduced the chances of its use among respondents (cOR=0.163; 95%CI:0.053-0.496, p≤0.001) and (cOR=0.213;95%CI:0.057-0.79, p≤0.021) respectively. However, respondents reported to smoke more than five times a day were 21 times most likely to use tobacco during pregnancy as compared to those who smoked less than five times a day (cOR=21.686; 95%CI:4.33-108.612, p≤0.001) and (cOR=16.345;95%CI:4.795-55.721,p≤0.001) respectively. On the contrary, the chances of tobacco use reduced with an increase in the age of the respondents. Thus, respondents aged 20 to 29 years were less likely to use tobacco during pregnancy (cOR=0.243; 95%CI: 0.066-0.901, p≤0.034).

### Adjusted analysis of factors associated with tobacco use among pregnant women

In adjusted logistic regression analysis, the chances of tobacco use during pregnancy reduced significantly with age; respondents aged 20 to 29 years had only 38.8% chances of tobacco use (aOR=0.388;95%CI:0.164-0.92, p≤0.032). Also, the study established that respondents who started smoking at age 20 to 29 years in a lifetime were 22 times most likely to use tobacco during pregnancy unlike those who aged 15 to 19 years (aOR=22;95%CI:4.688-103.235, p≤0.001). Similarly, respondents who started smoking at an age above 30 years were 38 times most likely to smoke during their current pregnancy (aOR=38.095;95CI:12.172-119.227, p≤0.001).

Regarding respondents’ opinion towards smoking during pregnancy, those who agreed that it is a sign of attaining maturity had 2.1 chances of smoking unlike those who were not sure (aOR=2.157; 95%CI: 1.077-4.316, p≤0.003). While the chances of using tobacco reduced by three times among respondents who disagreed that it is a sign of attaining maturity (aOR=3.455; 95%CI: 1.508-7.915, p≤0.003).

Furthermore, there were 3 times increased chances of tobacco use during pregnancy among respondents who agreed that it calm nerves unlike those who were not sure (aOR=3.829; 95%CI:1.837-7.983, p≤0.001). Meanwhile, respondents who reported disagreement that smoking calm nerves had 4 times reduced chances of tobacco use during pregnancy (aOR=4.412;95%CI:1.717-11.335,p≤0.002). Similarly, the study established that respondents whose opinion was that smoking help to have control over their health were 3 times more likely to use tobacco during pregnancy unlike those who were not sure (aOR=3.127; 95%:1.347-7.256, p≤0.008). On the other hand, respondents who used tobacco products accepted by their culture such as cigarettes had 75.5% reduced chances of using tobacco during pregnancy (aOR=0.245; 95%CI:0.077-0.775, p≤0.017).

Besides, the form of tobacco use had a significant association with its use during pregnancy, thus respondents who used smoking form of tobacco had 74.2% reduced chances of tobacco use during pregnancy (aOR=0.258;95%CI:0.104-0.637, p≤0.003). Finally, the study established that frequency of tobacco use determines its use during pregnancy. The evidence indicates that respondents who reported smoking more than five times a day had reduced chances of tobacco use in pregnancy (aOR=0.31;95%CI:0.126-0.76,p≤0.01).

## DISCUSSION

This study assessed the prevalence of tobacco use and associated risk factors among pregnant women in Maracha district west Nile region Uganda. It was found that more than a quarter of 39.2% of the respondents had used tobacco during pregnancy; of these, 19.1% had used tobacco in their lifetime. The prevalence of tobacco use obtained in this study is slightly higher than the 20% national prevalence reported among women 15 to 49 years that used tobacco products (UDHS, 2016). Also, the prevalence of tobacco use through second smoking was 35% among pregnant women in the West Nile district of Arua and the chances of tobacco use among pregnant women were twice (5%) the national average 2.3% (Apoto, 2011). On the contrary, Shannon (2018) revealed a very low prevalence of tobacco use among pregnant women in Tanzania (0.2%), Malawi (0.3%), and Burundi (0.3%). Similarly, the demographic health survey report showed a very low prevalence (2.6%) of tobacco use among 58,922 pregnant women 15-49 years in 54 low and middle-income countries from 2001 to 2012 (Caley et al., 2014, Murthy and Mishra, 2017). The high prevalence of tobacco use in this study is attributed to age at the start of tobacco use such as 5-15 years and duration of tobacco use for example 5-15 years (14.6%) and 26-35 years.

### Demographic characteristics associated with tobacco use among pregnant women

According to this study finding, the age of respondents was found statistically associated with tobacco use during pregnancy. Moreso, respondents aged 20 to 29 years were less likely to use tobacco during pregnancy compared to those aged 15 to 19 years. This finding contradicts with reports obtained in a study done in Nepal which found 19.2% of young aged women use tobacco during pregnancy, and 17% in Australia (Angelika et al., 2016) and 9.9% in Japan (Anu, 2013). However, Mitra (2012) reported 10% of higher aged women used tobacco during pregnancy in Bangladesh. Similarly, Pregnant women aged 25-34 years old smoked more cigarettes (58%) than those 24 years old or younger in America (Alexandria, 2014). The age variation of tobacco use obtained in this study is due to a lack of cultural values attached to smoking during pregnancy, and lack of culturally gazetted place and designated place for smoking that reduced the likelihood of tobacco use among pregnant women. Thus, maternal age has a negative influence on tobacco use during pregnancy in this study.

Furthermore, this study revealed that age at the start of smoking determines tobacco use during pregnancy. Hence, respondents who started tobacco use at the age of 30 years and above were 38 times most likely to use tobacco during pregnancy compared to those 15 to 19 years. Meanwhile, the study found that respondents who started smoking at ages 20 to 29 years in a lifetime were 22 times more likely to use tobacco during pregnancy unlike those aged 15 to 19 years. Contrary to this study finding, Rev.Latino (2010) reported tobacco use goes with age where most pregnant women that started smoking at 10 to 18 years were less likely to use tobacco during pregnancy unlike their counterparts 19 to 25 years (91 vs77.8%) in Brazil. This study finding is attributed to social norms like tobacco use is a sign of attaining maturity, having control over their life and health, and belief that tobacco smoking can help calm nerves, control moods, and alleviate stress. Therefore, tobacco use during pregnancy was found statistically associated with age at the start of smoking among pregnant women in Maracha County.

The study further revealed that the religious affiliation of respondents had a statistical relationship with tobacco use during pregnancy. Mostly, catholic respondents had 64.1% higher chances of using tobacco during pregnancy compared to those in other religious affiliations (p≤0.004). This study finding is contrary to the finding of Baron (2013) that reported different religious denominations to seem to be influencing tobacco use during pregnancy. For example, 9.2% of the women who smoked cigarettes during pregnancy in the Netherlands were nonreligious. Thus, the higher chances of tobacco use among catholic respondents could be due to their large number in terms of participation in the study.

Similarly, the study found further established that the marital status of the respondent had a statistical association with tobacco use during pregnancy at bivariate analysis. Married respondents had 76.5% chances of using tobacco during pregnancy unlike those who are single and separated (p-0.000). This contrasts with the report in Canada, where single mothers had more chances of tobacco use during pregnancy than their counterparts (Al-Sahab, 2010). Relatedly, centers for disease control and prevention report indicates that single mothers were four times more likely to smoke than married mothers during pregnancy in Pennsylvania (CDC, 2010). The higher chances of using tobacco among married respondents in this study are attributed to having smoker at home, lack of cultural punishment specifically for women who smoke during pregnancy and inadequate policy governing tobacco use since 26.1% of the respondents were not aware of policy that regulates tobacco use in Maracha county.

Even though the level of education did not have an impact on tobacco use at the multivariate level, findings at bivariate analysis revealed that respondent’s level of education was found statistically significantly associated with tobacco use during pregnancy. It was revealed that respondents who attained primary education had 65.4% chances of using tobacco during pregnancy compared to those who attained secondary and tertiary education (p-0.007). This is in line with results in Nepal where a 27% prevalence of tobacco use was reported among uneducated pregnant women compared to 20% among educated women Anu, 2013). In the same study, 34% of women living with partners who had attained primary level education used tobacco during pregnancy ( Anu, 2013). Moreso, In Bangladesh, illiterate women used tobacco during pregnancy more than their counterparts (Mitra, 2012). Thus, increasing the mother’s level of education decreases Mother’s Cigarette Smoking rates in the US (Alexandria, 2014).

According to this study finding, the likelihood of using tobacco during pregnancy was found higher among respondents who agreed that smoking is a sign of maturity (p-0.003) and a sign of wealth (p-0.034). This corresponds with finding obtained from a study conducted by Gillian et al. (2017) that showed maternal smoking was associated with communal smoking which is considered as a way of maintaining social cohesion (initiation, maintaining, and inheritance). And social norms were key socio-cultural factors in indigenous communities of High-Income countries of the US, Canada, Australia, and New Zealand. However, Bailey (2010) reported in a study done in Southern Appalachia that living with a smoker increases higher chances of influencing others to do the same. This study finding is attributed to the cultural acceptance of tobacco use in pregnancy (25.1%), and at least smoking multiple times a day (15.1%).

Furthermore, the study finding indicated that respondents who disagreed smoking make pregnant women feel they have a lot of control over their health and life were 3 times less likely to use tobacco during pregnancy unlike those who were not sure. However, this study finding contradicts with a report obtained in Nepal, where smoking cigarettes is often considered as a symbol of independence and freedom among women. It has become a fashion statement among people, especially urban women. While tobacco chewing is more socially acceptable than smoking, and among smokers, Bidi smoking is more common than cigarette smoking among the rural women (MOHP, 2012).

Besides, pregnant women who disagreed with smoking can help them calm nerves, control moods, and alleviates stress had 4 times reduced chances of using tobacco during pregnancy compared to those who were not sure (p-0.002). This is attributed to tobacco health-related problems like heart problem, 26.1%; cancer, 22.1%; tuberculosis, 22.6%; respiratory diseases, 4.0%; hypertension, and diabetes 1.5%; and reproductive health problems 0.5%.

### Socio-cultural factors associated with tobacco use among pregnant mothers

According to this study finding, having a culture that accepts the use of tobacco during pregnancy significantly influences its use among pregnant women. Moreso, respondents who indicated that their culture accepts tobacco use during pregnancy had 37.2% chances of smoking unlike six in ten who disagreed (p-0.002). Besides, the study established that the reasons culture accepts smoking in pregnancy influenced pregnant women to smoke (p-0.000). This is in agreement with results in the Appalachian population, where 54% of women who smoked during pregnancy were dully permitted/accepted to do so (Cottrell, 2007). Similarly, smoking cigarettes is often considered as a symbol of independence and freedom among women in Nepal.

Furthermore, the study established that the form of tobacco product determines its use during pregnancy. Thus, respondents who used cigarettes had reduced odds of smoking during pregnancy unlike those who use snuff. This is contrary to result obtained from a study that reported a 22% prevalence of tobacco use among pregnant women who used common forms of tobacco use such as cigarette smoking (15%) in Nepal. Other forms like smoking bids (5%), chewing tobacco (5%), and smoking pipe (4%) were also quite popular ( Anu, 2013).

The study further indicated that the product of tobacco influences its use during pregnancy. There was 37.2% chance of using tobacco during pregnancy among respondents who used smokeless products compared to those who used smoking (p-0.000). Relatedly, Ramesh et al. (2017) reported a substantial variation in smokeless tobacco use by ethnicity. For example, expectant mothers from Dalit and Aadibasi/Janajati ethnic groups were more likely to use smokeless tobacco than the upper caste group in Southern Terai of Nepal. Similarly, pregnant women from hill origin were more likely to use smokeless tobacco than Terai origin in Southern Terai of Nepal (Ramesh et al., 2017). Also, the study found that the frequency of use of tobacco per day had a significant association with its use during pregnancy. This is evidenced by the fact that respondents whose culture accepts smoking more than 5 times a day were less likely to use tobacco in pregnancy unlike those who smoke less than five times per day. This is in line with a global report which showed 72.5% of pregnant women smoked tobacco daily during pregnancy, unlike 27.5% who smoked occasionally (Shannon, 2018). The proportion of women who smoked daily and continued to smoke daily during pregnancy ranged from 30.6% in the European region to 79.6% in the Western Pacific region.

### Limitations

This was the first study to assess the prevalence of tobacco use among pregnant women in the study area; hence there was a challenge of getting local literature of the study area.

### Conclusions

The prevalence of tobacco use among pregnant women was found very high and it is significantly higher compared to report in other studies. This was associated with age at the start of tobacco use especially 30 years above and 20 to 29 years. Tobacco use at 20 to 29 years negatively influenced its use during pregnancy in this study, while smoking as a sign of attaining maturity, wealth, and having control over maternal health and helps to calm nerves had a significant association with tobacco use during pregnancy. Cultural acceptance of the use of tobacco products such as cigarettes increased its likelihood of use during pregnancy and smoking more than five times a day.

### Recommendations

The District Health Management Team (DHMT) together with cultural leaders should liaise in conducting health education and sensitization to raise the level of awareness about tobacco health related problems among pregnant mothers. The cultural leaders should revisit the acceptance of the use of tobacco among pregnant women in various forms. The government at all levels should enforce the tobacco control policy to eliminate the use of tobacco among pregnant women and the entire population at large. The policy should be stepped up through conducting an anti-tobacco campaign to raise the level of awareness about the existence of a policy that regulates tobacco use.

## Figures and Tables

**Table 1.** Demographic characteristics of the respondents.

Variable	Responses	Frequency n=199	Percentage	P-value
Maternal age	15-19 years	36	18.1	**0.021** **
	20-29 years	99	49.7	
	> 30 years	64	32.2	
Age at marriage	15-19 years	138	69.3	0.232
	20-29 years	56	28.1	
	> 30 years	5	2.5	
Age start smoking	15-19 years	36	18.1	**<0.001** **
	20-29 years	27	13.6	
	> 30 years	15	7.5	
	Not applicable	121	60.8	
Ethnic group	Lugbara	174	87.4	0.122
	Kakwa	9	4.5	
	Alur	3	1.5	
	Madi	9	4.5	
	Others	4	2	
Place of residence	Village	163	81.9	0.162
	Peri-urban	24	12.1	
	Urban	12	6	
Religion	Protestants	49	24.6	**0.004** **
	Catholics	126	63.3	
	Muslims	17	8.5	
	Others	7	3.5	
Marital status	Married	179	89.9	**<0.001** **
	Single	6	3	
	Separated	4	2	
	Divorced	6	3	
	Cohabiting	4	2	
Level of education	Primary	150	75.4	**0.007** **
	Secondary	27	13.6	
	Tertiary	10	5	
	University	2	1	
	Non-formal education	10	5	
Current pregnancy order	First pregnancy	32	16.1	**0.024** **
	Second pregnancy	39	19.6	
	Third pregnancy	30	15.1	
	Fourth pregnancy	36	18.1	
	Fifth pregnancy	23	11.6	
	More than five pregnancy	39	19.6	
Children ever born to a pregnant women	None	32	16.1	**0.004** **
	One child	48	24.1	
	Two children	22	11.1	
	Three children	36	18.1	
	Four children	22	11.1	
	Five children	10	5	
	Five children and above	29	14.6	
Number of children living	None	32	16.1	**0.024** **
	One child	47	23.6	
	Two children	35	17.6	
	Three children	31	15.6	
	Four children	19	9.5	
	Five children	15	7.5	
	Five children and above	20	10.1	
Family size	< 5 members	86	43.2	0.705
	> 5 members	113	56.8	
Occupation	Peasant farmer	149	74.9	0.109
	Civil servant	16	8	
	Business operator	23	11.6	
	Others	11	5.5	
Individual daily income	<9450	148	74.4	0.317
	>9450	51	25.6	
**Respondents opinion about Tobacco use**				
Sign of attaining maturity	Agree	50	25.1	**0.009** **
	Disagree	97	48.7	
	Not sure	52	26.1	
Sign of wealth	Agree	18	9	**0.034** **
	Disagree	137	68.8	
	Not sure	44	22.1	
To have control over their health and life	Agree	28	14.1	**0.02** **
	Disagree	130	65.3	
	Not sure	41	20.6	
Help calm nerves	Agree	43	21.6	**<0.001** **
	Disagree	119	59.8	
	Not sure	37	18.6	
	**Total**	**199**	**100**	

Source primary field data 2018

** statistically significant at P≤0.05.

**Table 2.** Demographic and cultural factors associated with tobacco use among pregnant Women.

Variable	P-value	COR 95%CI	P-value	AOR 95%CI
**Maternal age**	0.010			
15-19 years	0.847	0.881(0.243-3.198)	0.761	0.88(0.386-2004)
20-29 years	0.034	0.243(0.066-0.901)	0.032	0.388(0.164-0.92)
> 30 years		Reference		1
**Age start tobacco use**	0.000			
15-19 years	0.240	Reference		1
20-29 years	0.000	21.9(4.688-103.235)	0.000	22(4.688-103.235)
>30 years	0.000	38.09(12.172-119.227)	0.000	38.095(12.172-119.227)
**Sign of attaining maturity**				
Agree	0.650	1.216(0.522-2.837)	0.03	2.157(1.077-4.316)
Disagree	0.009	3.841(1.4-10.537)	0.003	3.455(1.508-7.915)
Not sure				1
**Control over their health**				
Agree	0.001	6.240(2.12-18.368)	0.008	3.127(1.347-7.256)
Disagree	9.951	0.959(0.256-3.59)	0.172	1.975(0.43-5.249)
Not sure		Reference		1
**Smoking calm nerves**	9.001			
Agree	0.002	3.838(1.615-9.12)	0.000	3.829(1.837-7.983)
Disagree	0.001	8.908(2.43-32.663)	0.002	4.412(1.717-11.335)
Not sure	0.020	Reference		1
**Socio-cultural factors**				
**Tobacco products accepted by a culture**				
Cigarette	0.013	20.250(1.878-218.39)	0.017	0.245(0.077-0.775)
Bidi	0.469	2.455(0.216-27.841)	0.293	0.269(0.023-3.101)
Snuff	0.003	23.707(2.912-193.025)	0.865	1.058(0.554-2.019)
**Form of tobacco use**	0.002			
Smoking	0.001	0.163(0.053-0.496)	0.003	0.258(0.104-0.637)
Smokeless	0.021	0.213(0.057-0.79)	0.037	1.068(0.568-2.008)
**Frequency of smoking per day**				
> 5 times	0.000	21.686(4.33-108.612)	0.01	0.31(0.126-0.76)
< 5 times	0.000	16.345(4.795-55.721)	0.056	1.887(0.983-3.624)

Source primary field data 2018.

COR; crude odds ratio and AOR; adjusted odds ratio.

## ABBREVIATIONS

CDC: Centers for Disease Control and Prevention
CTC: Centre for Tobacco Control
DHMT: District Health Management Team
DHS: Demographic Health Surveys
GATS: Global Adult Tobacco Survey
LMICS: Low- and Middle-Income Countries
MDLG: Maracha District Local Government
MOHP: Ministry of Health and Population
NDHS: Nepal Demographic Health Survey
NHS: National Health Survey
PNFP: Private Not For Profit
SHS: Second Hand Smoking
SLT: Smokeless Tobacco
SPSS: Statistical Package for Social Scientists
UDHS: Uganda Demographic Health Survey
The UK: United Kingdom
UNFPA: Uganda National Family Planning Association
The US: United States of America
UTCA: Uganda Tobacco Control Act
WHO: World Health Organization
